# Evaluation of sensory and safety quality characteristics of “high mountain tea”

**DOI:** 10.1002/fsn3.2923

**Published:** 2022-06-20

**Authors:** Cong‐ming Wang, Xiao Du, Cong‐ning Nie, Xiang Zhang, Xiao‐qin Tan, Qian Li

**Affiliations:** ^1^ Sichuan Agricultural University Chengdu China; ^2^ Tea Refining and Innovation Key Laboratory of Sichuan Province Chengdu China; ^3^ Chengdu Academy of Agriculture and Forestry Sciences Chengdu China; ^4^ Sichuan Academy of Agricultural Sciences Chengdu China

**Keywords:** aroma, heavy metals, high mountain tea, pesticide residues, taste

## Abstract

High mountain tea (HT) is widely acknowledged as an essential resource of high‐quality tea due to its adaptation to superior ecological environments. In this study, the sensory (aroma and taste) and safety (heavy metals and pesticide residues) characteristics of HT were characterized through sensory evaluation, gas chromatography–mass spectrometry (GC‐MS), liquid chromatography–mass spectrometry (LC‐MS), flavor activity value, and risk factor analysis. The results elucidated that the aroma sensory characteristics of HT were tender and green, accompanied by sweet and slight chestnut. A total of 8 aroma compounds were identified as the primary substances contributing to the unique aroma characteristics; the difference in the ratio of "green substances" and "chestnut substances" might be the reason for different aroma characteristics in HT and LT (low mountain tea). The taste sensory characteristics of HT were high in freshness and sweetness but low in bitterness and astringency. The high content of soluble sugar (SS), nonester catechins, sweet free amino acids, and low content of caffeine and tea polyphenols were the primary reasons for its taste characteristics. Low temperature stress might be the most fundamental reason for flavor characteristics formation in HT. Furthermore, the pollution risks of 5 heavy metals and 50 pesticide residues in HT were less than 1. The complex ecosystem and low chemical control level were speculated to be the primary reasons for the high safety quality of HT. Overall, these findings provide a more comprehensive understanding of quality characteristics and their formation mechanisms in HT.

## Graphical abstract

In this paper, the sensory (aroma and taste) quality and safety (heavy metals and pesticide residues) quality of high mountain tea (HT) were characterized. At the same time, it was proposed that "the different content and proportion of flavor substances is the direct reason for the flavor difference between HT and LT, and low temperature stress is the main reason for the flavor substance difference between HT and LT."

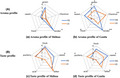

## 1 INTRODUCTION

In China, there is a common saying that "high mountain clouds make good tea," which means tea from high‐altitude areas usually has excellent quality. High mountain tea (HT) refers to the tea produced in high‐altitude mountainous areas, where the ecological environment is generally superior, with low temperature, less sunlight, and more clouds. Tea in these mountainous areas generally grows slowly but is rich in flavor substances (Luo et al., 2009; Nicole et al., 2019; Stilo et al., 2020; Wan, 2003). At the same time, due to the low yield of *Camellia*, and the shortage of labor force in mountainous areas, most of the HT areas adopt "natural agricultural method" cultivation, with less chemical fertilizer and pesticide application, and high quality and safety of tea (Liu et al., 2021; Zeng et al., 2011; Zheng et al., 2019). For decades, HT has also regarded as the "high‐end tea" and "luxury tea," with seldom access by ordinary people. However, the emerging living standards of people have increased the pursuit of quality and health, eventually increasing the concern toward HT consumption.

In the past, people's perception of "high mountain clouds make good tea" was just a basic knowledge toward tea consumption and production, or a common cognition of tea drinkers. In fact, the flavor characteristics of tea depend on the coordination of its components and their proportions (Zhang, Cao, et al., 2020). In the study of modern food flavor, most of them describe the flavor characteristics of tea quantitatively by constructing the "flavor map" and using the "contribution value" of flavor active components and analyze the correlation between sensory flavor and flavor components to evaluate the flavor characteristics and causes of tea. These methods have been used in the study of black tea, green tea, and yellow tea (Mario et al., 2020; Scharbert, Holzmann, & Hofmann, 2004; Schuh & Schieberle, 2006). However, there is no report on the study of HT quality by relevant methods.

Mengding Mountain, located in Sichuan Province, China, is one of the major tea‐producing areas in China. Shihua green tea produced in this area was once rated as one of the top ten famous teas in China. According to the survey, the main tea gardens in this area are mainly divided into two areas according to the altitude, one is a low mountain tea (LT) garden with an altitude of 600–800 m and the other is a HT garden with an altitude of 1000–1300 m. Since LT garden is the main tea‐producing area in this region, most of the studies on tea are concentrated in the low mountain area, while the research on the quality characteristics of HT and its difference with LT has not been reported so far. We believe that it is necessary to study the quality characteristics of HT. On the one hand, HT can be developed as a special tea resource, on the other hand, the development and utilization of HT can alleviate the current land pressure in LT areas and promote the sustainable development of tea.

Therefore, in the present study, HT (1000–1200 m) was selected as the representative test site and compared with LT (600–800 m) of this area to provide potential strategies for sustainable development of HT and tea. Further, the quality characteristics of high mountain ecological tea were analyzed from two aspects of flavor quality and safety quality using the modern food flavor method.

## 2 MATERIALS AND METHODS

### 2.1 Study area

**FIGURE 1:**
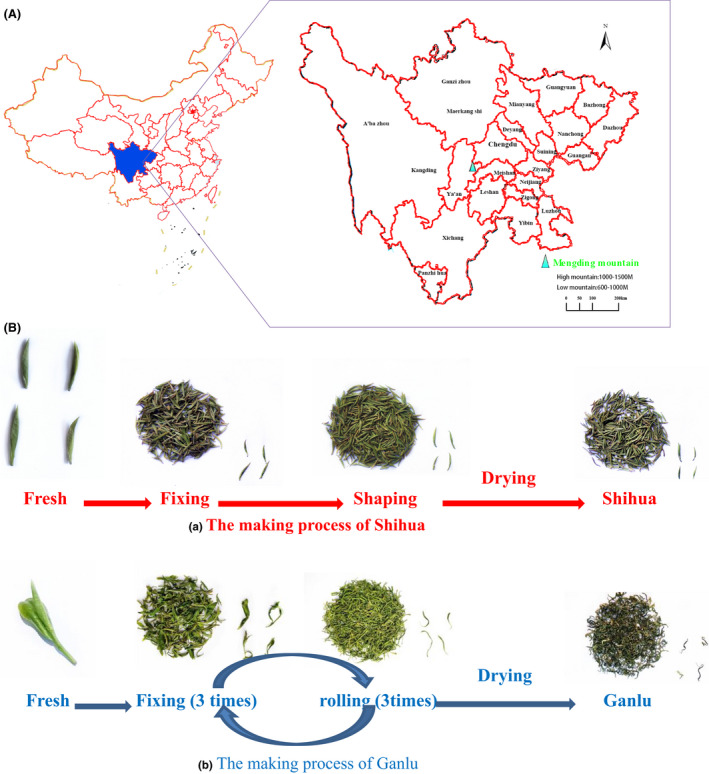
Location map of study area and treatment method of study materials. (a) Location map of study area; the sampling site is located in Mengding Mountain, Ya'an City, Sichuan Province, China. (b) The making process of Shihua and Ganlu. The map in (a) was drawn through “ArcGIS Desktop 10.5”

The experimental area was located in Mengding Mountain, Ya'an City, Sichuan Province, China (Figure 1a). According to the local lowest altitude (550 m) and the actual situation of local tea production, we divided the study area into LT garden (600–800 m above sea level) and HT garden (1000–1300 m above sea level). At the same time, we selected tea gardens with basically the same tea variety, tree age and tea garden cultivation, and management model in these two areas for comparison. Finally, a total of 6 Haoshan tea gardens and 7 LT gardens were selected. Table S1 summarizes the details of each tea garden.

### 2.2 Sample collection and processing

The fresh leaves of the "Sichuan middle‐leaf tea tree population" were collected from the above 13 areas in between the middle of March to the beginning of May 2020. Among them, 10.00 kg buds and 10.00 kg one bud with one leaf were collected in each area. All fresh tea leaves of the same batch were picked within one day, and the tea samples were prepared immediately. Of them, the buds (10.00 kg) were used to prepare the Shihua tea (1.90–2.00 kg) by the Shihua making process, as depicted in Figure 1b, (a), and the fresh leaves of one bud with one leaf (10.00 kg) were used to prepare the Ganlu tea (1.80–1.95 kg) by the Ganlu making process, as depicted in Figure 1b, (b). The detailed process parameters are summarized in Table S2. They were numbered, packed in composite aluminum bags, and frozen at −5°C.

### 2.3 Quantitative description evaluation

The solutions and the hydrolyzed infusions were scored by a trained team of 12 assessors (7 males, 5 females: age 23–27 years) from the Food College of Sichuan Agricultural University. The sensory evaluation room was independent, and each reviewer was separated by a simple board, with a space of 2 m. It was forbidden to communicate and discuss with each other (GB/T 18797‐2012, General Administration of Quality Supervision, Inspection, and Quarantine (AQSIQ) and the Standardization Administration of China (SAC), 2012).

The solution was sniffed and graded (5‐point scoring method) by the evaluation team trained in systematic sniffing (specific training methods refer to the previous reports of our group (Nie et al., 2019), including two parts, intensity training of aroma monomer and recognition training of mixed solution). All experiments were conducted in triplicate at room temperature (23 ± 1°C) using distilled water as a control check (flavor intensity was defined as 0).

The aroma evaluation method was conducted according to the method of our group (Nie et al., 2019) with minor modifications. The chosen aroma descriptors were green (reference standard aroma components are trans‐3‐hexenol), tender (nonanal), chestnut (3‐methylbutyraldehyde), sweet (phenylethanol), and fresh (trans‐2‐hexenal). The detailed description and explanation of flavor are summarized in Table S3. Two tea samples of 10.0 g were weighed into a triangular flask (250 ml volume), heated for 10 min in a water bath at 50 ± 1°C to ensure that the aroma diffuses into the bottle to saturate the space, and then immediately sniffed in a sensory panel room at 23 ± 1°C. In order to keep the temperature of aromatic solution constant, the sensory evaluation of aroma was carried out in a 50℃ water bath. Every compound was sniffed only once, and the sniffing trials were performed 10 min apart to overcome the memory effects of a single aroma compound.

The taste evaluation method was according to the method of Cao (Cao et al., 2019) and Li (Li et al., 2021) with minor modifications. The chosen taste descriptors were fresh (reference standard components are glutamate), sweet (sucrose), bitter (quinine), puckery (tannins), and acid (citrin). Around 3 g tea sample was infused in 150 ml of boiled water for 4 min in a special teacup. The tea infusion (60–70℃) was then poured immediately into a tea bowl and evaluated by the panel.

A 5‐point 0 (odorless)‐ 5 (strong) scoring method was selected for evaluation. The tea infusions were randomly offered to panelists after brewing. The solutions and the hydrolyzed infusions were scored at room temperature (23 ± 1°). They were asked to score the aroma qualities of green, tender, chestnut, sweet, fresh and the taste qualities of fresh, sweet, bitter, puckery, and acid on a scale from 0 to 5. The samples were analyzed in triplicate by each panelist, and the average score was used to draw the flavor profile.

### 2.4 Determination of aroma compounds

Extraction of aroma‑active components: the HS‐SPME method was selected to isolate the active aroma components; a tea sample (3.0 g) with 30 µg ethyl decanoate as an internal standard (10 mg/kg tea sample) was placed in an extraction bottle (15 ml volume). The sample was equilibrated in a thermostatic water bath for 10 min at 50°C, and then sampled for 30 min in the headspace (the head space temperature was still kept at 50℃). Afterward, SPME fiber was withdrawn and directly introduced to the GC‐MS (the temperature of sample for GC‐MS was 50℃), and the process was repeated thrice. The SPME fiber was 50/30 μm DVB/CAR/PDMS whose length was 1 cm (Sigma‐Aldrich), and the fiber penetration depth into the headspace was 2 cm.

GC‐MS instrument setup and analytical conditions: GC‐MS analyses were performed on an Agilent 7890A/5975C‐GC/MSD inert detector operating in EI mode in a 69.9 eV chromatographic column: capillary‐column chromatography DB‐5 ms (30 m × 250 μm × 0.25 μm). The sampling was manual, no shunt was used, the sample was kept at a constant temperature, and the injection port and GC‐MS direct interface temperatures were 250 and 280°C, respectively. Temperature programming: the column temperature was 50–250°C; the starting column temperature was 50°C which was held for 3 min, and then increased to 150°C at a rate of 2°C/min, held for 2 min, then increased to 250°C at a rate of 2.5°C/min and held for 4 min. Helium was used as a carrier gas at a flow rate of 1.0 ml/min. The ionic source temperature was 230°C; the quadrupole temperature was 150°C; and the scanning quality range was set at 20–700 amu.

Qualitative and quantitative analysis of aroma compounds: Qualitative analysis: the NIST standard spectral library (https://www.nist.gov/) and retention indices (RIs) from other literature were used to match ion mass spectra, and then the qualitative analysis of aroma components was completed. Quantitative analysis: the internal standard method was used to quantify the volatile flavor compounds in the tea aroma. Ethyl decanoate was selected as the internal standard (10 mg/kg tea sample).

### 2.5 Determination of taste compounds

Determination methods of total tea polyphenols, total amino acids, total caffeine, and total SS: 0.6 g (±0.001 g) of tea sample was weighed into a 150 ml conical flask, and 60 ml of boiling distilled water was added and immediately moved into a boiling water bath for 45 min (shaken every 10 min). After the extraction, it was immediately filtered under the condition of hot decompression. The filtrate was transferred into a 100 ml volumetric flask; the residue was washed with a small amount of hot distilled water for 2–3 times; and the filtrate was filtered into the volumetric flask. After cooling, the filtrate was diluted into 100 ml with distilled water. The methods were operated as specified in the following determination methods, and the content of each component in the sample was calculated according to the mass of the dry tea sample. The total amount of tea polyphenols was determined by Folin phenol method (GB/T8313‐2018; State Administration for Market Regulation (SAMR) and the Standardization Administration of China (SAC), 2018); the total amount of free amino acids was determined by Ninhydrin colorimetry (GB/T 8314‐2013; General Administration of Quality Supervision, Inspection, and Quarantine (AQSIQ) and the Standardization Administration of China (SAC), 2013a); the content of caffeine was determined by ultraviolet spectrophotometry (GB/T 8312‐2013; General Administration of Quality Supervision, Inspection, and Quarantine (AQSIQ) and the Standardization Administration of China (SAC), 2013b); and the total amount of SS was determined by anthrone colorimetry (Zhang, 2009).

Determination of catechin components: catechin contents were measured using high‐performance liquid chromatography (HPLC), following the national standard GB/T 8313‐2018 (State Administration for Market Regulation (SAMR) and the Standardization Administration of China (SAC), 2018) with some modifications. Briefly, 1.5 g (±0.001 g) of uniformly ground sample was weighed, added into 125 ml boiling water, extracted in boiling water bath for 45 min, pumped and filtered while hot, and finally, the volume was fixed to 250 ml. Later, the extract was filtered with a 0.45 μm water phase filter head (mixed cellulose ester filter membrane; Biosharp), and 2 ml of filtrate was collected for standby. Detector: Waters 600 pump HPLC (Waters 600; Waters), Waters empower chromatographic management system (Waters Empower 3, 2011), Waters 2489 UV detector (Waters 2498; Waters). Methods: the chromatographic column was Phenomenex Gemini C18 (Phenomenex Gemini 5u C18 110A, 250 mm × 4.6 mm; Phenomenex); the elution temperature was 20℃; the injection volume was 5 μl; the flow rate was 1 ml/min; and the detection wavelength was 278 nm. Phase A was 0.2% acetic acid solution, while phase B was pure acetonitrile. Elution procedure: the 100% phase A was kept for 10 min, and then changed from 100% phase A to 68% phase A and 32% phase B within 15 min, maintained this condition for 10 min, and then returned to 100% phase A.

Determination of amino acid composition: Free amino acid contents were measured using an automatic amino acid analyzer (model:L‐8900; supplier:Hitachi) following the national standard GB/T 30987‐2020 (State Administration for Market Regulation (SAMR) and the Standardization Administration of China (SAC), 2020). Briefly, 0.25 g (±0.0001 g) of uniformly ground sample was weighed into a 50 ml centrifuge tube. Later, 25 ml of boiling water was added and the sample was extracted in a boiling water bath for 45 min, then quickly cooled to room temperature, centrifuged at 4000 r/min for 10 min, and finally, the supernatant was drawn for testing. 5 ml of the supernatant was taken and added into 5 ml of 5% trifluoroacetic acid solution, centrifugated at 4℃ 7000 r/min for 20 min, passed through a 0.45 μm inorganic filter membrane, and then 20 μl of the test solution was sucked and injected into the automatic amino acid analyzer. Finally, the external standard method was used for quantitative analysis.

Each sample was measured thrice in parallel, and the average value was taken.

### 2.6 Determination of heavy metals in tea

Tea pretreatment: nitric acid perchloric acid wet digestion method was adopted, i.e., 1.0 g (±0.001 g) of the sample was weighed into a Teflon cup; 25 ml of mixed acid with a volume ratio of 5:1 (HNO_3_:HClO_4_) was added, placed in the fume hood, covered and soaked for 16h, heated with an electric heating plate; the temperature was adjusted to 150 ± 2℃; slowly digested until the residual 2–5 ml was colorless and transparent liquid; then it was removed and cooled. The deionized water was washed, filtered, and the volume was set to 25 ml.

Cr was detected by graphite furnace atomic absorption spectrometry (GB5009.123‐2014; National Health & Family Planning Commission of the People's Republic of China, 2014a); As was detected by liquid chromatography atomic fluorescence spectrometry (Haiguang LC‐AFS9530; GB5009.11‐2014; National Health & Family Planning Commission of the People's Republic of China, 2014b); Cd was detected by graphite furnace atomic absorption spectrometry (GB5009.15‐2014; National Health & Family Planning Commission of the People's Republic of China, 2014c); Pb was detected by graphite furnace atomic absorption spectrometry (GB5009.12‐2017; National Health & Family Planning Commission of the People's Republic of China, 2017a); and Cu was detected by flame atomic absorption spectrometry (GB5009.13‐2017; National Health & Family Planning Commission of the People's Republic of China, 2017b).

Detection parameters: the absorption wavelengths were 357.9 nm for Cr, 228.8 nm for Cd, 283.2 nm for Pb, and 324.7 nm for Cu, respectively. The lamp current was 10.0 mA; the spectral bandwidth was 0.7 nm; the high pressure was 207.00 v; the gas flow rate was 2.2 L/min; and the burner height was 7.0 mm.

### 2.7 Determination of pesticide residues in tea

Tea sample pretreatment: tea samples were extracted by ultrasonic with acetonitrile as extraction solvent, and were purified by Agilent Mega BE Carbon/NH_2_ Liquid filter tube. The pesticide residues, such as organophosphorus and organochlorine, were detected by gas chromatography (GC); insecticides with thermal instability or low vapor pressure, such as carbamates and N‐methyl carbamates, were detected by HPLC; imidacloprid and acetamiprid were detected by ultra‐performance liquid chromatography tandem mass spectrometry (LC‐MS); seven types of pyrethroids were detected by GC‐MS.

HPLC detection conditions: Agilent 1260 HPLC, Agilent C8 250 mm × 4.6 mm × 5 μm column was used; the column temperature was 42℃; Fluorescence detector: λex = 330 nm, λem = 465 nm; injection volume 10 μl; the mobile phase A was water and the mobile phase B was methanol; gradient elution procedure: 0.0–2.0 min, 85%–75% A; 2.0–6.5 min, 75% A; 6.5–10.5 min, 75%–60% A; 10.5–28.0 min, 60% A; 28.0–33.0 min, 60%–20% A; 33.0–35.0 min, 20% A; 35.0–37.0 min, 20%–0% A; 37.0–37.1 min, 0%–85.00% A; flow rate: 0.3 ml/min.

GC‐MS detection conditions: Agilent 7890b‐7000c gas chromatography tandem mass spectrometry was used; Agilent hp‐5ms 30 m × 250 μm × 0.25 μm capillary column was used; injection port temperature: 270℃; injection volume was 1 μl, no split flow injection; gradient heating program of column temperature box: after holding 80℃ for 2 min, 15℃/min was raised to 310℃ and kept for 5 min, with a total of 22.33 min; carrier gas N_2_ ≥ 99.999%, 1 ml/min constant current mode; mass spectrum interface temperature, 230℃; ion source temperature, 230℃; quadrupole temperature, 150℃; electron bombardment source voltage, 70 eV.

LC‐MS detection conditions: Agilent 1290‐6470 ultra‐high performance liquid chromatography tandem mass spectrometer, Agilent c1850 mm × 0.30 mm × 1.8 μm column. The injection volume was 1.0 μl; the mobile phase A was methanol and the mobile phase B was water (containing 0.1% formic acid). Gradient elution process: 0–1 min, 10% A; 1–6.6 min, 10%–80% A; 6.6–7.0 min, 80%–90% A; 7.0–7.10 min, 90%–98% A; 7.10–11.60 min, 98% A; 11.60–15.00 min, 98% A; flow rate, column temperature 30℃, electrospray ionization (ionization) source, positive ion mode, dry gas temperature 350℃, dry gas flow rate, nebulizer pressure, sheath gas temperature 300℃, sheath gas flow rate / the capillary voltage was 4000 V; scanning mode was DMRM.

### 2.8 Statistical analysis

The experimental results were expressed as means ± standard deviations (SD) of three parallel measurements. Correlations were calculated using partial least squares regression (PLSR). Statistical analyses were performed using SPSS (version 25.0, IBM126 Corp.).

## 3 RESULTS

### 3.1 Sensory characteristics of HT

Chivalrous tea senses mainly include aroma and taste senses of tea. The following mainly analyzes the aroma and taste characteristics of HT and its difference with LT.

#### 3.1.1 Aroma sensory characteristics of HT and its difference with LT

**FIGURE 2:**
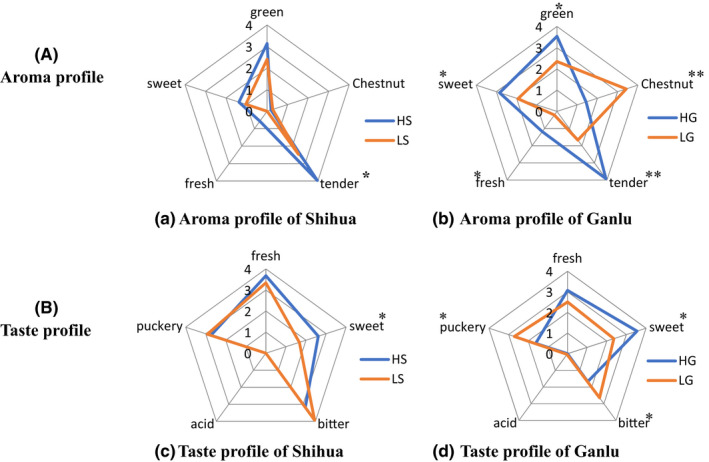
Flavor profile of different tea. (a) Location map of study area; the sampling site is located in Mengding Mountain, Ya'an City, Sichuan Province, China. (b) The making process of Shihua and Ganlu. The map in Figure 1a was drawn through “ArcGIS Desktop 10.5.” “HS,” High mountain Shihua, “LS,” Low mountain Shihua, “HG,” High mountain Ganlu, “LG,” Low mountain Ganlu. *p < .05; **p < .01. Pictures are drawn by Office 2019

The five aroma factors of HT and LT were scored by quantitative description analysis, and the flavor profile was drawn according to the average score (Figure 2a).

HT includes high mountain Shihua (HS) and high mountain Ganlu (HG). The aroma profile of HS showed a tender green predominance accompanied by a faint sweet, with average scores of 3.14, 0.19, 3.97, 0.57, 1.37 for green, chestnut, tender, fresh, and sweet, respectively (Figure 2a). Similarly, the aroma profile of HG showed tender, green, and sweet dominated accompanied by faint chestnut, with scores of 3.54, 1.44, 3.92, 1.18, 2.84 for green, chestnut, tender, fresh, and sweet, respectively (Figure 2b). These differences might attribute to the differences in the two processing modes and the complicated fabrication process with multiple rubbing twists and drying processes, affecting the internal components of tea and making its tea aroma profile more "plump."

LT also includes low mountain Shihua (LS) and low mountain Ganlu (LG). Compared with the LT, the aroma profile shape of HS was consistent with LS, with the only difference as the aroma profile of HS was slightly greater than LS (Figure 2a). This result indicated that HS had a higher aroma intensity than LS. Since Shihua is processed in a simple way to retain the original fresh leaf aroma, it was speculated that the differences in aroma profile were most likely due to the greater abundance of aroma substances in the fresh alpine leaves. HG exhibited quite different aroma profiles, such as high tender (+134.73%), green (+50.63%), sweet (+47.15%), and low chestnut (−58.02%) than LG (Figure 2b). Green versus tender was mainly derived from some alcohols, aldehydes, and esters with low boiling points, cis‐3‐hexenol, nonanal, and methyl salicylate (Nie et al., 2020). Chestnut aroma mainly comes from pyrrole and furan substances produced by Maillard reaction in the process of tea processing (Guo et al., 2018). Since HT and LT adopt the same processing method, the chestnut aroma substances produced by Maillard reaction should be basically the same. Therefore, we speculate that the main reason for the difference of chestnut aroma between them may be the masking or synergistic effect between different aroma types; this requires further analysis of their aroma components.

#### 3.1.2 Taste sensory characteristics of HT and its difference with LT

As for taste, the HS taste profile showed a predominance of fresh versus bitter, accompanied by a faint sweet, with the scores of 3.68, 2.61, 3.18, 0, 2.74 for fresh, sweet, bitter, acid, and puckery, respectively (Figure 2c). Similarly, the HG flavor profile showed a predominance of fresh versus sweet accompanied by an appropriate bitter degree as well as faint acid, with scores of 3.07, 3.54, 1.67, 0, 1.62 for fresh, sweet, bitter, acid, and puckery, respectively (Figure 2d).

Compared with LT, the sweet score of HS was 2.61, which was significantly higher than in LS (1.67; Figure 2c). In contrast, no significant differences were observed for other scores, such as fresh, bitterness, puckery, and acid. The SS in tea is the primary contributor to the sweetness of tea (Li et al., 2020), while the large temperature difference between day and night in the high mountains is conducive to the accumulation of SS in tea. This might be the main reason for higher sweet scores in HS than LS. Compared with LG, significant differences were observed in fresh, bitterness, puckery, and sweet scores, except for acid (Figure 2d). HG and LG showed two completely different taste characteristics. The scores of fresh and sweet in HG were 3.07 and 3.54, respectively, which were 22.31% and 50.00% higher than in LG. In contrast, the scores of puckery and bitterness in HG were significantly lower than in the shallow hill. Studies have proved that puckery and bitterness significantly reduce the taste of tea and consumers' preference (Yau & Huang, 2000). In this regard, HG's taste characteristics of high sweet, high fresh, and low bitterness are more easily accepted by consumers.

### 3.2 Content and activity of aroma compounds in tea

#### 3.2.1 Content of aroma compounds in tea

The content and proportion of tea aroma components directly determine the aroma sensory characteristics of tea. There are hundreds of aroma compounds in teas, including esters, alcohols, acids, ketones, and terpenes (Ho et al., 2015). In this study, a total of 38 aroma components were detected in two types of HT samples, including 13 alcohols, 3 ketones, 7 aldehydes, 4 esters, 8 hydrocarbons, and 3 heterocyclic compounds (Table 1).

**TABLE 1:** Content of aroma components, OAVs, and flavor description of different tea samples

No.	Category	Aroma components	Concentration (mg/kg)^a^				OAV^b^				Aroma description^c^
			HS	LS	HG	LG	HS	LS	HG	LG	
1	Alcohols	1‐Penten‐3‐ol	0.97 ± 0.08	0.30 ± 0.04	2.77 ± 0.25	2.41 ± 0.19	0.39	0.12	1.11	0.96	Butter, Fish, Green
2		1‐Pentanol	0.19 ± 0.02	0.17 ± 0.01	0.08 ± 0.02	0.08 ± 0.01	0.04	0.03	0.02	0.02	Balsamic, Fruit, Green
3		(Z)‐3‐hexen‐1‐ol	4.01 ± 0.27	3.36 ± 0.28	1.75 ± 0.14	0.34 ± 0.03	20.04	16.80	8.75	1.70	Grass, Green Fruit, Green Leaf
4		1‐Hexanol	–	–	0.23 ± 0.02	0.04 ± 0.05	Null	Null	0.90	0.17	Banana, Flower, Grass
5		2‐Ethylhexanol	2.62 ± 0.33	1.48 ± 0.19	2.28 ± 0.30	1.14 ± 0.17	Null	Null	Null	Null	Green, Rose
6		Benzyl alcohol	3.46 ± 0.45	2.95 ± 0.27	2.09 ± 0.24	1.58 ± 0.13	0.63	0.54	0.38	0.29	Boiled Cherries, Moss, Roasted Bread, Rose
7		Linalool oxide I	0.75 ± 0.05	0.65 ± 0.03	1.30 ± 0.11	0.58 ± 0.03	125.60	108.80	216.00	96.80	Floral
8		Linalool	1.37 ± 0.10	1.51 ± 0.14	2.29 ± 0.18	1.06 ± 0.09	912.00	1004.80	1529.60	704.00	Coriander, Floral, Lavender, Lemon, Rose
9		Linalool oxide II	1.00 ± 0.11	0.58 ± 0.02	1.92 ± 0.08	0.62 ± 0.14	166.40	96.80	320.00	104.00	Floral
10		Phenethyl alcohol	1.94 ± 0.17	1.15 ± 0.09	1.55 ± 0.05	1.99 ± 0.14	1.94	1.15	1.55	1.99	Fruit, Honey, Lilac, Rose, Wine
11		dl‐menthol	0.73 ± 0.12	0.75 ± 0.04	0.75 ± 0.05	0.60 ± 0.08	2.44	2.50	2.50	2.02	Mint, Cool
12		Nerolidol	0.75 ± 0.06	–	1.97 ± 0.24	–	50.30	Null	131.04	Null	Rose, Keiskei, Apple
13		Geraniol	0.94 ± 0.14	–	1.05 ± 0.11	0.94 ± 0.05	125.18	Null	139.78	125.95	Geranium, Lemon Peel, Passion Fruit, Peach, Rose
14	Ketone	6‐Methyl‐5‐hepten‐2‐one	0.96 ± 0.08	–	0.43 ± 0.02	–	9.62	Null	4.30	Null	Fruit, Fresh
15		A‐ionone	0.26 ± 0.05	0.28 ± 0.01	0.55 ± 0.10	0.74 ± 0.05	639.36	695.52	1373.76	1844.64	Violet, Fruit, Wood
16		Geranylacetone	0.72 ± 0.02	–	1.72 ± 0.25	0.25 ± 0.02	12.01	Null	28.63	4.09	Apple, Banana
17	Aldehyde	Valeraldehyde	–	–	2.45 ± 0.33	5.14 ± 0.38	Null	Null	24.47	51.42	Almond, Bitter, Malt, Oil, Pungent
18		Hexanal	1.59 ± 0.18	2.38 ± 0.31	4.53 ± 0.51	3.85 ± 0.46	317.95	476.93	905.47	770.00	Apple, Fat, Fresh, Green
19		Heptaldehyde	0.88 ± 0.06	2.45 ± 0.15	4.53 ± 0.17	2.31 ± 0.18	28.32	78.93	146.04	74.47	Citrus, Fat, Green, Nut
20		Benzaldehyde	1.28 ± 0.03	1.02 ± 0.04	1.45 ± 0.08	1.09 ± 0.11	4.26	3.41	4.84	3.62	Bitter Almond, Burnt Sugar, Cherry, Roasted Pepper
21		2,4‐Heptadienal	–	–	0.73 ± 0.078	0.56 ± 0.01	Null	Null	207.36	159.96	Fat, Nut
22		Nonanal	1.65 ± 0.12	1.89 ± 0.13	2.85 ± 0.27	1.87 ± 0.13	471.99	539.14	813.64	535.19	Fat, Floral, Green, Lemon
23		Β‐cyclocitral	0.74 ± 0.11	0.72 ± 0.04	0.86 ± 0.05	0.82 ± 0.14	147.92	143.77	171.42	164.51	Fresh fruit, Camphor
24	Ester	Hexyl acetate	1.16 ± 0.30	–	1.18 ± 0.25	0.82 ± 0.12	29.03	Null	29.55	20.39	Apple, Banana, Grass, Herb, Pear
25		Methyl salicylate	1.58 ± 0.19	0.39 ± 0.04	0.72 ± 0.08	–	26.41	6.45	11.95	Null	Almond, Caramel, Peppermint, Sharp
26		(Z)‐3‐hexenyl hexanoate	1.30 ± 0.07	0.28 ± 0.03	1.06 ± 0.11	0.24 ± 0.03	13.03	2.78	10.61	2.40	Fruit, Prune
27		Benzyl benzoate	0.79 ± 0.04	0.68 ± 0.03	0.81 ± 0.06	0.68 ± 0.02	0.79	0.68	0.81	0.68	Balsamic, Herb, Oil
28	Hydrocarbon	dl‐Limonene	0.59 ± 0.03	–	–	0.48 ± 0.02	1.19	Null	Null	0.95	Orange
29		A‐pinene	–	–	0.20 ± 0.01	1.09 ± 0.18	Null	Null	1.66	9.10	Cedarwood, Pine, Sharp
30		A‐farnesene	–	–	0.31 ± 0.02	0.61 ± 0.03	Null	Null	0.62	1.22	Boiled Vegetable, Floral, Wood
31		A‐caryophyllene	–	–	0.57 ± 0.02	2.38 ± 0.30	Null	Null	3.56	14.88	Wood
32		3‐Carene	–	–	0.44 ± 0.02	1.79 ± 0.14	Null	Null	3.38	13.76	Pine, Wood
33		Myrcene	0.63 ± 0.04	1.11 ± 0.02	0.73 ± 0.01	1.30 ± 0.01	44.93	79.49	51.84	92.82	Balsamic, Fruit, Geranium, Herb, Mus
34		Longifolene	0.35 ± 0.04	1.73 ± 0.15	2.99 ± 0.18	5.26 ± 0.32	Null	Null	Null	Null	Pine
35		N‐heptadecane	–	–	0.10 ± 0.01	0.80 ± 0.03	Null	Null	Null	Null	Sweet, Floral, Preserves
36	Heterocyclic	2‐Acetylfuran	–	–	0.60 ± 0.01	0.73 ± 0.02	Null	Null	0.01	0.01	Butter, Floral, Fruit, Green Bean
37		2‐Pentylfuran	–	–	0.52 ± 0.02	0.59 ± 0.03	Null	Null	108.00	122.40	Butter, Caramel
38		2‐Acetyl pyrrole	0.89 ± 0.07	0.77 ± 0.07	0.66 ± 0.02	0.73 ± 0.04	0.01	0.01	0.01	0.01	Bread, Cocoa, Hazelnut, Licorice, Walnut

^a^ In order to better explain the aroma characteristics of different tea samples, the representative tea samples with the most suitable aroma profile (*n* = 1) were selected for GC‐MS detection. The standard deviation is the standard deviation of three repeated measurements.

^b^ OAV = C/OT, *C* is the average concentration of a single volatile component, OT is the odor threshold of the same volatile component. The references of OT: Leffingwell & Associates Database (http://www.leffingwell.com/). Compilations of odor threshold values in air, water, and other media, and compilations of flavor threshold values in water and other media (2011 editions)—by van Gemert (http://www.thresholdcompilation.com/).

^c^ The source of aroma description: FEMA GRAS data base (https://www.femaflavor.org/). “–” means not detected or below the detection limit. “Null” means there is no relevant threshold or OAV cannot be calculated.

A total of 28 aroma substances were detected in HS samples, among which the high content aroma components mainly include (z)‐3‐hexen‐1‐ol, benzyl alcohol, 2‐ethylhexanol, phenyl alcohol, nonanal, valeraldehyde, and methyl salicylate, with the contents of 4.01, 3.46, 2.62, 1.94, 1.65, 1.59, and 1.58 mg/kg, respectively. A total of 37 aroma compounds were detected in HG samples, among which the high content aroma components mainly include hexanal, heptaldehyde, longifolene, nonanal, 1‐penten‐3‐ol, valeraldehyde, and linalool, with the contents of 4.53, 4.53, 2.99, 2.85, 2.77, 2.45, and 2.29 mg/kg, respectively. Among these aroma compounds, there are four aroma substances common to HS and Hg, including (z)‐3‐hexene‐1‐alcohol, benzyl alcohol, glutaraldehyde, and nonanal. It is speculated that they are the main aroma components constituting the aroma quality of HT.

The content and species of volatile components in HT were higher than in LT (Shihua or Ganlu; Table 1). In HT and LT, the aroma compounds with large difference (*p* < .05) and high content (content >1 mg/kg) were: (z)‐3‐hexen‐1‐ol, 2‐ethylhexanol, linalool, linalool oxide II, nerolidol, geranylacetone, heptaldehyde, methyl salicylate, (z)‐3‐hexenyl hexanoate, alpha pinene, alpha caryophyllene, and 3‐carene. These aroma components might dominate the difference in aroma sense between high mountain and LT. The alcohol contents in HT, such as (z)‐3‐hexen‐1‐ol, nerolidol, and linalool were higher than in LT, while the contents of terpenoids, such as alpha pinene, alpha caryophyllene, and 3‐carene were lower than in LT. In aroma description, most of these alcohols presented green, flower, and fruit aroma, while most of the terpenoids presented woody aroma. The presence of these woody aroma compounds might develop other aromas in tea by masking the expression of green.

2‐Pentylfuran with caramel flavor is often considered as one of the primary aroma attributes influencing the formation of chestnut (Ryoko & Kenji, 2014; Zhu et al., 2018). In this study, the content of 2‐pentylfuran in HG and LG was 0.52 and 0.59 mg/kg, respectively, with no significant difference (Table 1). Therefore, it was speculated that the reason behind HG having the characteristics of high green and low chestnut aroma is that it contains a large number of “green” alcohols substances that mask the expression of “chestnut” and “woody” substances. In LG, the expression of green alcohol compounds was weakened by more woody compounds, while the expression of chestnut compounds was promoted by woody compounds.

#### 3.2.2 Activity values of aroma compounds in tea

In the study of tea flavor, odor activity value (OAV) is often used to characterize the contribution of aroma compounds to the overall aroma (Scharbert, Jezussek, & Hofmann, 2004). It is generally recognized that the aroma components with OAV >1 have a significant contribution to the overall aroma of tea samples, and the higher is the OAV value, the higher will be the contribution to the overall aroma. In this study, a total of 28 aroma compounds with OAV >1 were detected in HT, including 21 aroma compounds in HS and 27 aroma compounds in HG (Table 1). Of them, 8 aroma compounds, including linalool (912.00/1529.60), alpha ionone (639.36/1373.76), nonanal (471.99/813.64), hexanal (317.95/905.47), linalool oxide II (166.40/320.00), 2,2‐Dimethyl‐6‐methylenecyclohexane‐1‐carbaldehyde (147.92/171.42), linalool oxide I (125.60/216.00), and geraniol (125.18/139.78) had OAV >100 both in HS and HG. In contrast, four compounds, including 2‐acetyl pyrrole (0.01/0.01), 1‐pentanol (0.04/0.02), benzyl alcohol (0.63/0.38), and benzyl benzoate (0.79/0.81), had OAV <1 both in HS and HG. These aroma components might not be the key components of HT aroma characteristics.

In sensory evaluation, HS aroma was mainly described as tender and green, accompanied by faint sweet, while HG aroma was mainly described as tender, green, and sweet, accompanied by faint chestnut (Figure 2b). According to the OAV of aroma substances and their aroma description, linalool aroma was described as "coriander, floral, lavender, lemon, rose," alpha ionone as "violet, fruit, wood," nonanal as "fat, floral, green, lemon," hexanal as "apple, fat, fresh, green," linalool oxide I and linalool oxide II as "floral," 2‐dimethyl‐6‐methylenecyclohexane‐1‐carbaldehyde as "fresh fruit, camphor," geraniol as "geranium, lemon peel, passage fruit, peach, rose." The interaction of these aroma substances with each other forms the unique aroma characteristics in HT.

### 3.3 Content and contribution value of taste compounds in tea

The taste of tea is characterized by its taste components, and the content and proportion of these substances have a profound impact on the taste of tea soup (Wang & Ruan, 2009). Therefore, the taste components in HT samples were further detected and analyzed to explore the taste characteristics and causes of HT.

#### 3.3.1 Content of taste compounds in tea

As summarized in Table 2, only three taste substances were significantly different in HS and LS, namely SS, phenylalanine (Phe), and gallocatechin gallate (GCG), while six were found in Ganlu, namely caffeine (CA), SS, glutamine (Gln), Phe, arginine (Arg), and epigallocatechin (EGC). This phenomenon showed a great relationship between the two processing methods and the source of raw materials. Firstly, the raw materials (one shoot and one leaf) of Ganlu had a longer growth and development time, which was more influenced by the environment. Secondly, the processing Ganlu was more complex, which further amplified the gap between these two.

**TABLE 2:** Contents of main taste substances in different teas

Taste substances	Concentration(mg/g)		Significance	Concentration(mg/g)		Significance^a^	Taste description^b^
	HS(*n* = 6)	LS(*n* = 7)		HG(*n* = 6)	LG(*n* = 7)		
TPs	240.42 ± 16.13	243.4 ± 9.50	NS	200.65 ± 13.17	220.28 ± 10.86	NS	Bitter, astringent
AA	52.55 ± 11.28	50.9 ± 6.93	NS	47.43 ± 9.15	42.75 ± 6.83	NS	Fresh, sweet
CA	46.67 ± 1.97	49.8 ± 3.01	NS	22.84 ± 2.64	43.85 ± 1.12	*	Bitter
SS	40.16 ± 2.64	25.63 ± 1.75	*	53.44 ± 1.73	29.10 ± 2.23	*	Sweet
The	22.45 ± 0.72	21.97 ± 2.41	NS	15.59 ± 1.1	15.32 ± 0.82	NS	Fresh, sweet
Asp	5.42 ± 0.24	5.16 ± 0.17	NS	3.1 ± 0.15	2.01 ± 0.08	NS	Fresh, slightly sour
Asn	2.45 ± 0.27	2.33 ± 0.14	NS	0.43 ± 0.08	0.49 ± 0.02	NS	Fresh, slightly sour
Glu	6.05 ± 0.19	5.77 ± 0.42	NS	3.04 ± 0.27	2.42 ± 0.08	NS	Fresh, slightly sour
Gln	2.03 ± 0.08	2.43 ± 0.06	NS	2.15 ± 0.19	1.30 ± 0.07	*	Fresh, slightly sour
Ser	1.05 ± 0.03	0.96 ± 0.08	NS	0.77 ± 0.27	0.53 ± 0.11	NS	Sweet
Ala	0.36 ± 0.01	0.35 ± 0.03	NS	0.32 ± 0.18	0.20 ± 0.05	NS	Sweet
Phe	0.82 ± 0.01	2.2 ± 0.04	*	0.14 ± 0.03	0.73 ± 0.04	**	Bitter
Arg	1.07 ± 0.04	1.12 ± 0.02	NS	0.71 ± 0.02	1.88 ± 0.19	*	Bitter
EGCG	113.72 ± 10.84	121.73 ± 8.24	NS	91.6 ± 9.68	109.92 ± 11.26	NS	Bitter, astringent
GCG	4.04 ± 0.17	7.58 ± 0.98	*	12.69 ± 1.37	14.47 ± 0.81	NS	Bitter
ECG	22.02 ± 5.57	28.38 ± 2.34	NS	28.96 ± 3.39	31.16 ± 1.99	NS	Bitter, astringent
EGC	8.82 ± 1.58	6.83 ± 1.17	NS	27.59 ± 1.68	15.62 ± 2.64	*	Bitter, slightly sweet
EC	6.12 ± 0.95	5.99 ± 0.57	NS	8.85 ± 0.87	10.51 ± 0.57	NS	Bitter, slightly sweet
C	1.41 ± 0.06	1.4 ± 0.03	NS	1.38 ± 0.15	1.12 ± 0.24	NS	Bitter, slightly sweet

Abbreviations: AA, Amino acids; Ala, Alanine; Arg, Arginine; Asn, Asparagine; Asp, Aspartic acid; C, Catechin; CA, Caffeine; EC, Epicatechin; ECG, Epicatechin gallate; EGC, Epigallocatechin; EGCG, Epigallocatechin gallate; GCG, Gallocatechin gallate; Gln, Glutamine; Glu, Glutamic acid; Phe, Phenylalanine; Ser, Serine; SS, Soluble sugar; The, Theanine; TPs, Tea polyphenols.

^a^ Significance: NS means no significant difference between samples; *Significantly different at *p* ≤ .05; **Significantly different at *p* ≤ .01.

^b^ The source of taste description: FEMA GRAS data base (https://www.femaflavor.org).

The primary distinct taste substances between HT and LT were bitter and sweet substances. For instance, CA, Phe, Arg, and EGC of bitter components in HG were significantly lower than in LG, while SS of sweet components were significantly higher than in LG (*p* < .05). The differences in these taste substances led to different sensory profiles of these tastes (Figure 2a).

#### 3.3.2 Contribution of taste compounds in tea

**FIGURE 3:**
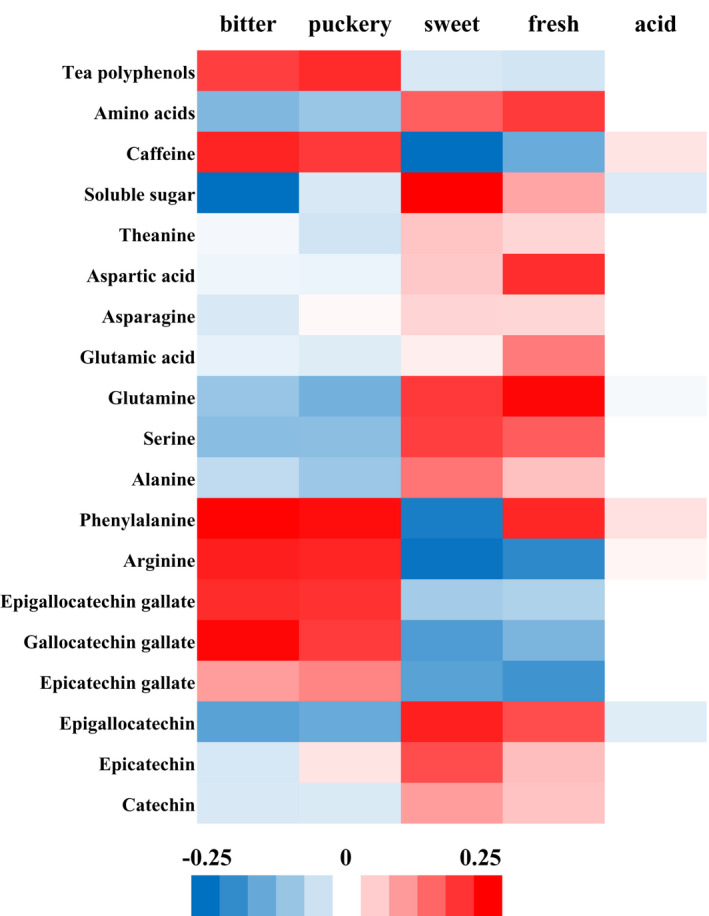
Correlation between main taste substances and sensory characteristics of HT. Here, we use the standardized coefficient of partial least squares regression (PLSR) to analyze the contribution of 20 tea flavor substances to 5 tea flavor attributes

The complex structure of the primary taste substances of tea, such as tea polyphenols and SS, with a nonunified threshold, limits the taste activity value (TAV) determination. Therefore, the standardized coefficient of PLSR was employed to analyze the contribution of 20 taste substances to 5 taste attributes of tea, and the results are depicted in Figure 3. The color depth represents the contribution; red is the positive contribution and blue is the negative contribution. It can be seen from the figure that polyphenols, caffeine, ester catechins (EGCG, ECG, GCG), and some bitter amino acids (Phe, Arg) contributed to the bitterness and puckery of tea. Similarly, the SSs and some amino acids (Asp, Gln, Ser) contributed to the fresh sweetness of tea. The contribution of theanine to fresh was weaker than some free amino acids, such as aspartic acid and glutamine. This might have happened due to its high threshold. Besides, few substances related to sour taste were observed, mainly because green tea has almost no sour taste.

### 3.4 Safety quality characteristics of HT

Besides the sensory quality, the tea quality also includes the safety quality. The primary regulatory factors of tea safety quality include the types and contents of pesticide residues, harmful heavy metals, and microorganisms. In this study, 5 toxic heavy metals and 50 types (see Table S4 for details) of pesticide residues in different tea samples of HT and LT were detected, and the results are summarized in Table 3.

**TABLE 3:** Contents and pollution index of heavy metals and pesticide residues in tea

Risk elements	Contents (mg/kg)				Limit value^a^ (mg/kg)	Pollution index^b^			
	HS (*n* = 6)	LS (*n* = 7)	HG (*n* = 6)	LG (*n* = 7)		HS	LS	HG	LG
Heavy metal									
Cr	1.650 ± 0.712ab	1.724 ± 0.294a	1.940 ± 0.481a	1.331 ± 0.093b	5.000	0.330	0.345	0.388	0.266
As	0.074 ± 0.017b	0.070 ± 0.024b	0.033 ± 0.007a	0.060 ± 0.017b	2.000	0.037	0.035	0.017	0.030
Cd	0.065 ± 0.005c	0.026 ± 0.005a	0.035 ± 0.001b	0.057 ± 0.008c	1.000	0.065	0.026	0.035	0.057
Pb	0.708 ± 0.015a	1.835 ± 0.501b	0.554 ± 0.008a	0.716 ± 0.247a	5.000	0.142	0.367	0.111	0.143
Cu	14.083 ± 1.547a	17.989 ± 0.271b	13.881 ± 0.462a	17.731 ± 1.385b	60.000	0.235	0.300	0.231	0.296
Pesticide residues									
Imidacloprid	0.001 ± 0a	0.005 ± 0.002b	0.001 ± 0a	0.008 ± 0.004b	0.500	0.001	0.005	0.002	0.012
Acetamiprid	–	0.002 ± 0a	–	0.015 ± 0.001b	10.000	Null	Null	Null	0.002
Carbendazim	–	0.006 ± 0a	0.009 ± 0b	0.021 ± 0.003c	5.000	Null	0.001	0.004	Null
Chlorfenapyr	–	**–**	–	0.470 ± 0.094	20.000	Null	Null	Null	0.024
Buprofezin	–	–	–	0.500 ± 0.137	10.000	Null	Null	Null	0.050
Chlorpyrifos	0.086 ± 0.015b	0.170 ± 0.021d	0.130 ± 0.009c	0.042 ± 0.011a	1.000	0.086	0.170	0.130	0.042
Cypermethrin	0.046 ± 0.002a	0.045 ± 0.009a	0.047 ± 0.009a	0.072 ± 0.010b	20.000	0.002	0.002	0.002	0.004
Lamda‐cyhalothrin	0.044 ± 0.006a	0.041 ± 0.012a	0.080 ± 0.024b	0.130 ± 0.032c	15.000	0.003	0.003	0.005	0.009
Pyridaben	‐	0.010 ± 0. 003a	0.009 ± 0. 002a	0.890 ± 0.271b	5.000	Null	0.002	0.002	0.178
Bifenthrin	‐	0.039 ± 0. 006a	0.026 ± 0.005b	0.904 ± 0.316c	5.000	Null	0.008	0.005	0.380

^a^ Limit value: The limit value of heavy metals in tea comes from China National Standard: limit of pollutants in food (GB 2762‐2017); the pesticide residue limit value of tea comes from the Chinese national standard: maximum residue limit of pesticide in food (GB 2763‐2019).

^b^ Pollution index = w/ws, w is the content of element, ws is the limit value of element.

#### 3.4.1 Evaluation of five toxic heavy metals in HT

The average content of five heavy metals in HT was far lower than the maximum limit value of the corresponding standard. The contents of Cr, As, Cd, Pb, and Cu in HS were 67.0%, 96.3%, 93.5%, 85.8%, and 76.5% lower than the limit value, respectively, while the contents of Cr, As, Cd, Pb, and Cu in HG were 61.2%, 98.35%, 96.5%, 88.9%, and 76.9% lower than the limit value, respectively. This indicated that all HT were not polluted by heavy metals, and the risk of heavy metal pollution in HT was very low.

Compared with LT, HT contained more Cr, As, and Cd. Of these three heavy metal elements, the average pollution index of Cr was the largest, reaching 0.388, while the pollution index of the other two heavy metals was smaller (<0.1). Therefore, CR was speculated to be the most toxic heavy metal element in HT at present. Hence, the control of Cr should be strengthened in the future during the production and processing of HT.

#### 3.4.2 Evaluation of 50 pesticide residues in HT

We tested 50 pesticide residues in all HT samples, and the results showed that only 7 pesticide residues were detected in all HT samples. Of them, 4 were detected in HS and 7 in HG, and all of them were found to be well below the corresponding minimum limit values (Table 3). Taking the pesticide chloropyrifos, having a relatively large pollution index in HT as an example, its pollution index in HS and HG was only 0.086 and 0.130, respectively, which was much lower than its limit pollution index 1, suggesting high safety factor of HT.

Compared with LT, the agroforestry species and contents of the HT samples were all lower than in the LT samples. For instance, only four agroforestry residues were detected in HS, whereas eight were detected in LS. This could be explained as the pesticide application was much less in the HT area with a rich ecosystem and a cold winter season where infestation occurs less frequently.

## 4 DISCUSSION

### 4.1 What are the reasons for the difference in flavor and quality between alpine and LT?

#### 4.1.1 The direct cause

The flavor of food is determined by the flavor substances contained in the food itself, so is tea (Chaturvedula & Prakash, 2011; Peigen et al., 2014; Wang et al., 2019; Yamanishi et al., 1970). The flavor of tea is directly related to the content and proportion of flavor substances, and the difference in the content and proportion of these flavor substances is the most direct reason for the difference in flavor quality between HT and LT.

The sensory evaluation elucidated that the aroma of high mountain green tea was mainly tender and green, accompanied by a certain amount of sweet and weak chestnut, while the aroma of low mountain green tea was mainly chestnut, accompanied by a certain amount of tender, green, and sweet. The detection of related components and their contents by GC‐MS demonstrated consistent component content and dose–effect relationship (Table 1). The measured data illustrated that the alcohol contents with fruity and green flavor in HT were indeed higher than in LT, which was the main reason for its unique aroma. Meanwhile, tea aroma is not only related to the content and ratio of tea components but also shows synergistic, masking, and tone‐changing effects among the components (Liao et al., 2008; Wang et al., 2020; Yang et al., 2013; Zhu et al., 2017, 2021), reflecting the differences in tea flavor characteristics and sensory experience. Previous research has indicated that under the same OAV, the aroma components of floral fragrance could be easily masked by the aroma of other fragrances (Nie et al., 2019). Based on the aroma components detection results, it was speculated that the difference in the ratio (G/C) of "green substances" and "chestnut substances" might be the other reason for different aroma characteristics in HT and LT. For instance, since the G/C value of HG was relatively large, many green and fruit‐flavorings, such as (Z)‐3‐hexen‐1‐ol, benzyl alcohol, linalool, and nerolidol, might have covered up the expression of chestnut substances, thus presenting high green and low chestnut. However, the G/C value of LG was relatively small. Hence, the green substances with low content could not cover up the chestnut substances, thus presenting high chestnut.

**FIGURE 4:**
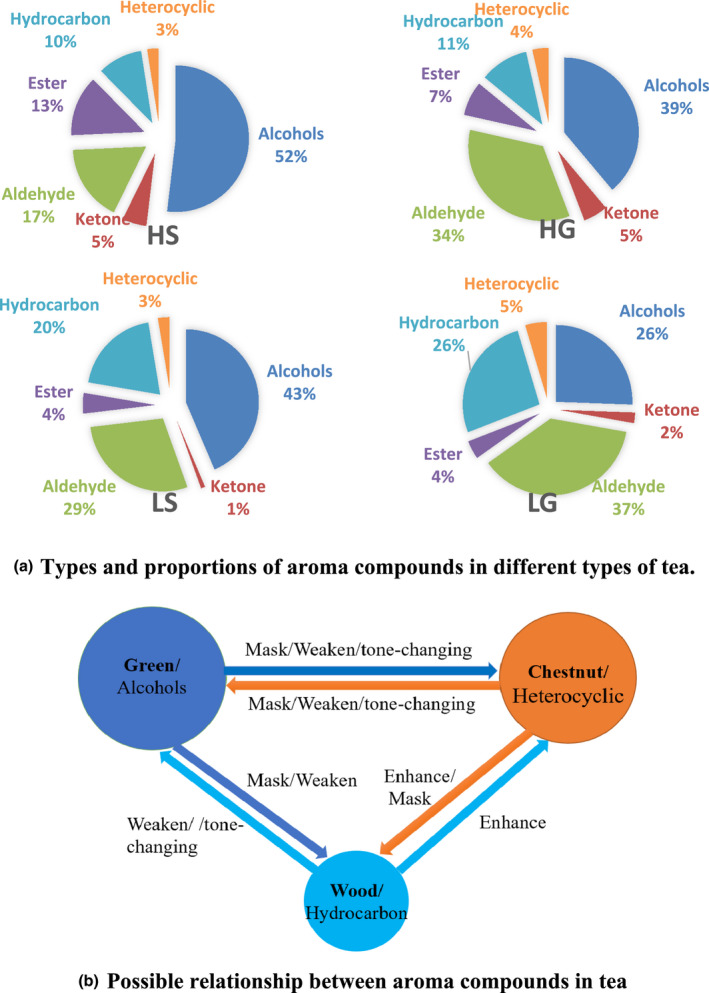
The proportion of aroma compounds in HT and LT and the possible relationship between them. Pictures are drawn by Office 2019

Additionally, the content of hydrocarbons in LT was relatively high, and most of these hydrocarbons were "wood." These wood substances might have an offsetting effect on green substances and a promoting effect on chestnuts, thereby promoting the expression of chestnut substances. Besides the aroma substance composition and proportion relationship, the interaction between aroma components (masking/offset/enhancement) was also an important factor that affected HT aroma (Figure 4). However, further study on the interaction between the aroma components is warranted to explore the mechanism behind the formation of high mountain green tea scents.

#### 4.1.2 The root cause

Tea flavor quality directly depends on the content and proportion of tea flavor substances, and the formation of tea flavor substances is a very complex process, such as variety difference, planting age difference, field cultivation management level difference and ecological environment difference (Selena et al., 2019; Zhang, Li, et al., 2020), which will have a significant impact on tea flavor quality. In this study, the varieties, tree age, cultivation management, and processing methods of HT and LT are basically the same (Table S1). Therefore, we focus on the impact of ecological environment on the quality difference between HT and LT.

The accumulation of flavor substances in HT is related to the high mountain environment, such as low temperature, cloudy, and diffuse light. Under such conditions, the late germination of tea bud and the slow growth of tender leaves are conducive to the accumulation of inclusion and optimization of the ratio (Niwa & Yamamoto, 1977). The results showed that the annual average temperature of HT tea garden was 2–3.5℃ lower than LT tea garden, the annual sunshine time was 400–550 h less, and the bud and leaf production period was 15–36 days more (the data came from the internal investigation of the local meteorological bureau and the research group). The conditions of low temperature and less light are conducive to nitrogen metabolism and increase the accumulation of amino acids in tea, which are the main flavor contributing substances in tea. At the same time, low temperature and less light can inhibit carbon metabolism to a certain extent, and then reduce the synthesis of bitter and astringent substances such as tea polyphenols and caffeine (Jayasekera et al., 2014).

Generally speaking, the temperature drops by 0.5℃ for every 100m increase in the average altitude, so HT are more vulnerable to the influence of low temperature stress. Under low temperature stress, polysaccharides in tea leaves will be transformed into SSs such as sucrose and glucose, which can increase the concentration of cell fluid and enhance the cold resistance of plants (William et al., 1992). At the same time, due to the large temperature difference between day and night in high altitude areas, SS accumulates in large quantities in tea leaves. SS is an important contribution to the sweetness of tea, and "sweetness" is also one of the biggest characteristics that distinguish HT from LT (Figure 2b). In addition, low temperature stress can also promote the accumulation of glycosides in tea (Zhao et al., 2020). When tea leaves are bitten by pests, these glycosides will be hydrolyzed and produce a large number of aroma substances, such as nerolidol, geraniol, linalool, benzyl alcohol, and phenylethyl alcohol (Bonaventure et al., 2011; Cai et al., 2014; Han & Chen, 2002). Most of these substances are fruity, floral, and green, and it is these substances that give HT unique aroma characteristics.

## 5 CONCLUSION

In this paper, the sensory (aroma and taste) quality and safety (heavy metals and pesticide residues) quality of HT were characterized. At the same time, it was proposed that "the different content and proportion of flavor substances is the direct reason for the flavor difference between HT and LT, and low temperature stress is the main reason for the flavor substance difference between HT and LT." The results of this study give us a more comprehensive understanding of HT and provide potential development strategies for the high quality and sustainable development of tea. Our research also provides a method for similar research.

The flavor of food depends on the flavor substances contained in the food itself, so does tea. The flavor of tea is directly related to the content and proportion of its flavor substances (direct cause), and the content and proportion of these flavor substances are related to the variety, cultivation and management, processing methods, soil nutrients, climate, and environment of tea (root cause). Our research focuses on the former (direct cause) rather than the latter (root cause). For example, through sensory evaluation, we found that the sweetness of HT is higher than that of LT. Then we analyze and discuss which flavor substances produce this sensory feature, as for how these flavor substances are produced, although important, is not the focus of this paper. The formation of flavor components and proportion in tea is a complex problem, which cannot be completely studied in a single experiment. However, for completeness and preciseness, we try to discuss the root causes of tea flavor formation according to the existing literature and small‐scale experimental data, but this is not the main task of this study. The main purpose of this study is to better understand the difference of quality characteristics between HT and LT and provide reference for us to better develop and utilize HT and similar commercial crop resources with special value.

## CONFLICT OF INTEREST

The authors declare that they do not have any conflict of interest.

## ETHICAL APPROVAL

In the sensory evaluation part of this study, trained evaluators were used to conduct necessary sensory evaluation on tea samples. Except for the above parts, this study does not include other work with human or animal. This study was approved by the local ethics committee of China: "Sichuan Regional ethics review committee of traditional Chinese Medicine," and all methods were carried out in accordance with relevant guidelines and regulations. Informed consent was obtained from all subjects before their participation in the study.

## Supporting information

Table S1‐S4

## References

[fsn32923-bib-0001] Bonaventure, G. , VanDoorn, A. , & Baldwin, I. T. (2011). Herbivore‐associated elicitors: FAC signaling and metabolism. Trends in Plant Science, 16, 294–299. 10.1016/j.tplants.2011.01.006 21354852

[fsn32923-bib-0002] Cai, X. , Sun, X. , Dong, W. , Wang, G. , & Chen, Z. (2014). Herbivore species, infestation time, and herbivore density affect induced volatiles in tea plants. Chemoecology, 24, 1–14. 10.1007/s00049-013-0141-2

[fsn32923-bib-0003] Cao, Q. Q. , Zou, C. , Zhang, Y.‐H. , Du, Q.‐Z. , Yin, J.‐F. , Shi, J. , Xue, S. , & Xu, Y.‐Q. (2019). Improving the taste of autumn green tea with tannase. Food Chemistry, 277, 432–437. 10.1016/j.foodchem.2018.10.146 30502167

[fsn32923-bib-0004] Chaturvedula, V. S. P. , & Prakash, I. (2011). The aroma, taste, color and bioactive constituents of tea. Journal of Medicinal Plants Research, 5(11), 2110–2124.

[fsn32923-bib-0006] General Administration of Quality Supervision, Inspection and Quarantine (AQSIQ) and the Standardization Administration of China (SAC) . (2012). General requirement of the tea sensory test room, GB/T 18797‐2012. China Standard Publishing House.

[fsn32923-bib-0007] General Administration of Quality Supervision, Inspection and Quarantine (AQSIQ) and the Standardization Administration of China (SAC) . (2013). Tea‐determination of caffeine content, GB/T 8312‐2013. China Standard Publishing House.

[fsn32923-bib-0008] General Administration of Quality Supervision, Inspection and Quarantine (AQSIQ) and the Standardization Administration of China (SAC) . (2013). Tea‐Determination of free amino acids content, GB/T 8314‐2013. China Standard Publishing House.

[fsn32923-bib-0009] Guo, X. , Song, C. , Ho, C. , & Wan, X. (2018). Contribution of l–theanine to the formation of 2, 5–dimethyl pyrazine, a key roasted peanutty flavor in Oolong tea during manufacturing processes. Food Chemistry, 263, 18–28. 10.1016/j.foodchem.2018.04.117 29784304

[fsn32923-bib-0010] Han, B. Y. , & Chen, Z. M. (2002). Composition of the volatiles from intact and mechanically pierced tea aphid‐tea shoot complexes and their attraction to natural enemies of the tea aphid. Journal of Agricultural and Food Chemistry, 50, 2571–2575. 10.1021/jf010681x 11958624

[fsn32923-bib-0011] Han, W. Y. , Selena, A. , Wei, C. L. , Orians, C. M. , & Landi, M. (2020). Editorial: Responses of tea plants to climate change: From molecules to ecosystems. Frontiers in Plant Science, 11, 204. 10.3389/fpls.2020.594317 33329660 PMC7732546

[fsn32923-bib-0012] Ho, C. T. , Zheng, X. , & Li, S. M. (2015). Tea aroma formation. Food Science and Human Wellness, 4, 9–27. 10.1016/j.fshw.2015.04.001

[fsn32923-bib-0013] Jayasekera, S. , Kaur, L. , Molan, A. , Garg, M. L. , & Moughan, P. J. (2014). Effects of season and plantation on phenolic content of unfermented and fermented Sri Lankan tea. Food Chemistry, 152, 546–551. 10.1016/j.foodchem.2013.12.005 24444973

[fsn32923-bib-0015] Li, Q. , Jin, Y. , Jiang, R. , Xu, Y. , Zhang, Y. , Luo, Y. , Huang, J. , Wang, K. , & Liu, Z. (2020). Dynamic changes in the metabolite profile and taste characteristics of Fu brick tea during the manufacturing process. Food Chemistry, 344, 128576. 10.1016/j.foodchem.2020.128576 33223295

[fsn32923-bib-0016] Li, Q. , Jin, Y. , Jiang, R. , Xu, Y. , Zhang, Y. , Luo, Y. , Huang, J. , Wang, K. , & Liu, Z. (2021). Dynamic changes in the metabolite profile and taste characteristics of Fu brick tea during the manufacturing process. Food Chemistry, 344, 128576. 10.1016/j.foodchem.2020.128576 33223295

[fsn32923-bib-0017] Liao, Y. C. , Tuong, H. B. , Imre, B. , & Fabien, R. (2008). Temporal changes in aroma release of Longjing tea infusion: Interaction of volatile and nonvolatile tea components and formation of 2‐butyl‐2‐octenal upon aging. Journal of Agricultural and Food Chemistry, 56, 2160–2169. 10.1021/jf073132l 18298066

[fsn32923-bib-0018] Liu, S. , Yao, X. , Zhao, D. , & Lu, L. (2021). Evaluation of the ecological benefits of tea gardens in Meitan County, China, using the InVEST model. Environment, Development and Sustainability, 23(5), 7140–7155. 10.1007/s10668-020-00908-6

[fsn32923-bib-0019] Luo, J. , Jin, L. X. , Han, Y. W. , & Wen, H. H. (2009). The research of the relationship between altitude and the quality of tea at Mengshan tea producing areas in Sichuan province. Journal of Southwest China Normal University (Natural Science Edition), 34, 122–127.

[fsn32923-bib-0020] Mario, F. , Sally, Q. , Guodong, W. , Xiaogen, Y. , & Peter, S. (2020). Characterization of the key odorants in a high‐grade chinese green tea beverage (*Camellia sinensis*; Jingshan Cha) by means of the sensomics approach and elucidation of odorant changes in tea leaves caused by the tea manufacturing process. Journal of Agricultural and Food Chemistry, 68, 5168–5179. 32251584 10.1021/acs.jafc.0c01300

[fsn32923-bib-0022] National Health and Family Planning Commission of the People's Republic of China . (2014). Determination of Cr in food, GB5009.123‐2014. China Standard Publishing House.

[fsn32923-bib-0023] National Health and Family Planning Commission of the People's Republic of China . (2014). Determination of As in food, GB5009.11‐2014. China Standard Publishing House.

[fsn32923-bib-0024] National Health and Family Planning Commission of the People's Republic of China . (2014). Determination of Cd in food, GB5009.15‐2014. China Standard Publishing House.

[fsn32923-bib-0025] National Health and Family Planning Commission of the People's Republic of China . (2017). Determination of Cu in food, GB5009.13‐2017. China Standard Publishing House.

[fsn32923-bib-0026] National Health and Family Planning Commission of the People's Republic of China . (2017). Determination of Pb in food, GB5009.12‐2017. China Standard Publishing House.

[fsn32923-bib-0027] Kfoury, N. , Scott, E. R. , Orians, C. M. , Ahmed, S. , Cash, S. B. , Griffin, T. , Matyas, C. , Stepp, J. R. , Han, W. , Xue, D. , Long, C. , & Jr Robbat, A. . (2019). Plant‐climate interaction effects: Changes in the relative distribution and concentration of the volatile tea leaf metabolome in 2014–2016. Frontiers in Plant Science, 10, 1518. 10.3389/fpls.2019.01518 31824541 PMC6882950

[fsn32923-bib-0028] Nie, C. N. , Zhong, X.‐x. , He, L. , Gao, Y. , Zhang, X. , Wang, C.‐m. , & Du, X. (2019). Comparison of different aroma‐active compounds of Sichuan dark brick tea (*Camellia sinensis*) and Sichuan Fuzhuan brick tea using gas chromatography‐mass spectrometry (GC–MS) and aroma descriptive profile tests. European Food Research and Technology, 245, 1963–1979. 10.1007/s00217-019-03304-1

[fsn32923-bib-0029] Nie, C. N. , Gao, Y. , Du, X. , Bian, J.‐L. , Li, H. , Zhang, X. , Wang, C.‐M. , & Li, S.‐Y. (2020). Characterization of the effect of cis‐3‐hexen‐1‐ol on green tea aroma. Scientific Reports, 10(1), 1–15. 10.1038/s41598-020-72495-5 32968179 PMC7511323

[fsn32923-bib-0030] Niwa, C. , & Yamamoto, R. (1977). Changes of climate and tea production in highland area. Chagyo Kenkyu Hokoku (Tea Research Journal), 1977(46), 29–37. 10.5979/cha.1977.46_29

[fsn32923-bib-0031] Peigen, Y. , Soo‐Lee, Y. A. , Mei‐Yin, L. , & Weibiao, Z. (2014). Identifying key non‐volatile compounds in ready‐to‐drink green tea and their impact on taste profile. Food Chemistry, 155, 9–16. 10.1016/j.foodchem.2014.01.046 24594147

[fsn32923-bib-0032] Ryoko, B. , & Kenji, K. (2014). Characterization of the potent odorants contributing to the characteristic aroma of Chinese green tea infusions by aroma extract dilution analysis. Journal of Agricultural and Food Chemistry, 62, 8308–8313. 10.1021/jf502308a 25088347

[fsn32923-bib-0033] Scharbert, S. , Holzmann, N. , & Hofmann, T. (2004). Identification of the astringent taste compounds in black tea infusions by combining instrumental analysis and human bioresponse. Journal of Agricultural and Food Chemistry, 52, 3498–3508. 10.1021/jf049802u 15161222

[fsn32923-bib-0034] Scharbert, S. , Jezussek, M. , & Hofmann, T. (2004). Evaluation of the taste contribution of theaflavins in black tea infusions using the taste activity concept. European Food Research and Technology, 218, 442–447.

[fsn32923-bib-0035] Schuh, C. , & Schieberle, P. (2006). Characterization of the key aroma compounds in the beverage prepared from Darjeeling black tea: Quantitative differences between tea leaves and infusion. Journal of Agricultural and Food Chemistry, 54, 916–924. 16448203 10.1021/jf052495n

[fsn32923-bib-0036] Selena, A. , et al. (2019). Environmental factors variably impact tea secondary metabolites in the context of climate change. Frontiers in Plant Science, 10, 939. 31475018 10.3389/fpls.2019.00939 PMC6702324

[fsn32923-bib-0037] Shu, K. , Kenji, K. , Hideki, M. , Andrea, H. , & Thomas, H. (2006). Molecular and sensory studies on the Umami taste of Japanese green tea. Journal of Agricultural and Food Chemistry, 54, 2688–2694. 10.1021/jf0525232 16569062

[fsn32923-bib-0038] State Administration for Market Regulation (SAMR) and the Standardization Administration of China (SAC) (2018). Determination of total polyphenols and catechins content in tea, GB/T8313‐2018. China Standard Publishing House.

[fsn32923-bib-0039] State Administration for Market Regulation (SAMR) and the Standardization Administration of China (SAC) (2020). Determination of free amino acids in plant, GB/T 30987‐2020. China Standard Publishing House.

[fsn32923-bib-0040] Stilo, F. , Tredici, G. , Bicchi, C. , Robbat, A. , Morimoto, J. , & Cordero, C. (2020). Climate and processing effects on tea (*Camellia sinensis* L. Kuntze) metabolome: Accurate profiling and fingerprinting by comprehensive two‐dimensional gas chromatography/time‐of‐flight mass spectrometry. Molecules, 25(10), 2447. 10.3390/molecules25102447 PMC7288030 32456315

[fsn32923-bib-0042] Wan, X. C. (2003). Tea biochemistry. China Agriculture Press.

[fsn32923-bib-0043] Wang, K. , & Ruan, J. (2009). Analysis of chemical components in green tea in relation with perceived quality, a case study with Longjing teas. International Journal of Food Science & Technology, 44, 2476–2484.

[fsn32923-bib-0044] Wang, M. , Ma, W.‐J. , Shi, J. , Zhu, Y. , Lin, Z. , & Lv, H.‐P. (2020). Characterization of the key aroma compounds in Longjing tea using stir bar sorptive extraction (SBSE) combined with gas chromatography‐mass spectrometry (GC–MS), gas chromatography‐olfactometry (GC‐O), odor activity value (OAV), and aroma recombination. Food Research International, 130, 108908. 10.1016/j.foodres.2019.108908 32156355

[fsn32923-bib-0045] Wang, Y. , Zheng, P.‐C. , Liu, P.‐P. , Song, X.‐W. , Guo, F. , Li, Y.‐Y. , Ni, D.‐J. , & Jiang, C.‐J. (2019). Novel insight into the role of withering process in characteristic flavor formation of teas using transcriptome analysis and metabolite profiling. Food Chemistry, 272, 313–322. 10.1016/j.foodchem.2018.08.013 30309549

[fsn32923-bib-0046] William, S. , Othieno, C. O. , & Carr, M. K. V. (1992). Climate and weather variability at the tea research foundation of Kenya. Agricultural and Forest Meteorology, 61, 219–235. 10.1016/0168-1923(92)90051-5

[fsn32923-bib-0047] Yamanishi, T. , Michiko, N. , & Yoichi, N. (1970). Studies on the flavor of green tea: Part VIII. Further investigation of flavor constituents in manufactured green tea. Agricultural and Biological Chemistry, 34(4), 599–608. 10.1080/00021369.1970.10859655

[fsn32923-bib-0048] Yang, Z. Y. , Baldermann, S. , & Watanabe, N. (2013). Recent studies of the volatile compounds in tea. Food Research International, 53, 585–599.

[fsn32923-bib-0050] Yau, N. J. N. , & Huang, Y. J. (2000). The effect of membrane‐processed water on sensory properties of oolong tea drinks. Food Quality and Preference, 11, 331–339. 10.1016/S0950-3293(00)00007-0

[fsn32923-bib-0051] Zeng, Q. G. , et al. (2011). Relationship between high‐quality tea and the geological background in Qionglai mountains. Agricultural Science & Technology, 12, 908–913.

[fsn32923-bib-0052] Zhang, L. , Cao, Q. , Granato, D. , Xu, Y. , & Ho, C. (2020). Association between chemistry and taste of tea: A review. Trends in Food Science & Technology, 101, 139–149. 10.1016/j.tifs.2020.05.015

[fsn32923-bib-0053] Zhang, Q. W. , Li, T. , Wang, Q. , LeCompte, J. , Harkess, R. L. , & Bi, G. (2020). Screening tea cultivars for novel climates: Plant growth and leaf quality of *Camellia sinensis* cultivars grown in Mississippi, United States. Frontiers in Plant Science, 11, 280. 10.3389/fpls.2020.00280 32231677 PMC7083152

[fsn32923-bib-0054] Zhang, Z. Z. (2009). Experimental course of tea biochemistry. China Agricultural Publishing House.

[fsn32923-bib-0055] Zhao, M. Y. , Zhang, N. , Gao, T. , Jin, J. , Jing, T. , Wang, J. , Wu, Y. , Wan, X. , Schwab, W. , & Song, C. (2020). Sesquiterpene glucosylation mediated by glucosyltransferase UGT91Q2 is involved in the modulation of cold stress tolerance in tea plants. New Phytologist, 226, 362–372. 10.1111/nph.16364 31828806

[fsn32923-bib-0056] Zheng, R. , Zhan, J. , Liu, L. , Ma, Y. , Wang, Z. , Xie, L. , & He, D. (2019). Factors and minimal subsidy associated with tea farmers’ willingness to adopt ecological pest management. Sustainability, 11(22), 6190. 10.3390/su11226190

[fsn32923-bib-0057] Zhu, J. C. , Chen, F. , Wang, L. Y. , Niu, Y. W. , & Xiao, Z. B. (2017). Evaluation of the synergism among volatile compounds in oolong tea infusion by odour threshold with sensory analysis and E‐nose. Food Chemistry, 221, 1484–1490. 10.1016/j.foodchem.2016.11.002 27979119

[fsn32923-bib-0058] Zhu, J. C. , Niu, Y. W. , & Xiao, Z. B. (2021). Characterization of the key aroma compounds in Laoshan green teas by application of odour activity value (OAV), gas chromatography‐mass spectrometry‐olfactometry (GC‐MS‐O) and comprehensive two‐dimensional gas chromatography mass spectrometry (GC×GC‐qMS). Food Chemistry, 339.127628. 10.1016/j.foodchem.2020.127628 33152893

[fsn32923-bib-0059] Zhu, Y. , et al. (2018). Identification of key odorants responsible for chestnut‐like aroma quality of green teas. Food Research International, 108, 74–82. 29735103 10.1016/j.foodres.2018.03.026

