# Supplementary material for: Evaluation of sensory and safety quality characteristics of “high mountain tea”
Source: Food Sci Nutr. 2022 Jun 20;10(10):3338–54. doi: 10.1002/fsn3.2923 (PMC9548367; doi:10.1002/fsn3.2923)
Supplement: Supplementary file 1 — Table S1‐S4 [file FSN3-10-3338-s001.docx]

**Evaluation of sensory and safety quality characteristics of "High mountain tea"**

Cong‑ming Wang^1^, Xiao Du^1*^, Cong‑ning Nie^2^, Xiang Zhang^3^, Xiao-qin Tan ^1^, Qian Li ^1^

^1^ Sichuan Agricultural University, No. 211 Huimin Road, Wenjiang District, Chengdu 610000, Sichuan, China

^2^ Chengdu Academy of agriculture and Forestry Sciences, No. 200 Nongke Road, Gongping street, Wenjiang District, Chengdu 610000, Sichuan, China

^3^ Sichuan Academy of Agricultural Sciences, No. 20 jingjusi Road, Jinjiang District, Chengdu 610000, Sichuan, China

* Corresponding author, email: [10669@sicau.edu.cn](mailto:10669@sicau.edu.cn)

**Table** **S1. Basic information of sampling points**

| spot | Geographic information | | | Tea information | | | | Tea garden management information | | | | Background value of tea garden soil^f^ | | | | |
| --- | --- | --- | --- | --- | --- | --- | --- | --- | --- | --- | --- | --- | --- | --- | --- | --- |
|  | altitude（m） | longitude° | latitude° | varieties | age^a^  (a) | height（m） | range（m） | fertilizer^b^ | pesticides^c^ | trim^d^ | plant^e^ | organic matter  (g/kg) | total nitrogen  (g/kg) | available phosphorus  (mg/kg) | available potassium  (mg/kg) | pH |
| HT1 | 1316±13 | 103.04 | 30.08 | There are one or more tea varieties in different tea gardens, but the tested varieties are all "Sichuan middle-leaf tea tree population" | 35-40 | 1.1±0.1 | 0.9±0.2 | 4 | 2 | 2 | D | 56.1±11.7 | 5.1±1.7 | 35.1±5.4 | 127.8±6.5 | 4.5±0.2 |
| HT2 | 1036±10 | 103.08 | 29.93 |  | 35-40 | 1.1±0.1 | 1.0±0.1 | 3 | 5 | 2 | D | 39.5±3.2 | 4.0±0.2 | 39.5±1.4 | 118.5±4.1 | 4.2±0 |
| HT3 | 1298±18 | 103.06 | 30.07 |  | 35-40 | 0.9±0.1 | 0.8±0.1 | 3 | 2 | 2 | D | 38.5±5.8 | 4.7±0.2 | 31.6±2.5 | 100.6±3.9 | 4.9±0.2 |
| HT4 | 1181±11 | 102.96 | 30.03 |  | 35-40 | 1.5±0.3 | 0.8±0.6 | 5 | 2 | 1 | S | 50.7±29.4 | 3.3±2.5 | 48.5±10.5 | 126.2±20.5 | 4.1±0.3 |
| HT5 | 1254±12 | 102.95 | 29.97 |  | 30-40 | 1.1±0.2 | 0.8±0.1 | 3 | 3 | 2 | D | 48.7±3.4 | 6.5±0.6 | 33.5±3.0 | 90.1±5.4 | 4.9±0.1 |
| HT6 | 1291±14 | 102.85 | 30.24 |  | 25-35 | 1.0±0.1 | 0.7±0.2 | 3 | 2 | 2 | D | 39.8±11.4 | 6.2±1.8 | 42.4±7.1 | 97.4±6.1 | 4.4±0.1 |
| LT1 | 610±5 | 103.34 | 30.22 |  | 35-40 | 1.1±0.1 | 0.9±0.2 | 3 | 5 | 3 | D | 29.6±1.7 | 3.6±0.1 | 36.2±0.8 | 119.4±2.6 | 5.0±0.1 |
| LT2 | 651±7 | 102.94 | 29.98 |  | 35-40 | 1.2±0.1 | 1.0±0.1 | 3 | 4 | 3 | D | 40.6±6.2 | 5.2±1.3 | 44.9±9.1 | 126.8±2.7 | 4.6±0.1 |
| LT3 | 600±9 | 103.15 | 29.98 |  | 30-40 | 0.9±0.1 | 0.8±0.1 | 5 | 7 | 2 | D | 60.7±4.8 | 5.6±1.1 | 41.4±2.0 | 131.9±1.5 | 4.1±0.1 |
| LT4 | 745±7 | 103.12 | 30.12 |  | 25-30 | 1.2±0.2 | 0.8±0.3 | 4 | 3 | 3 | D | 37.7±4.4 | 4.1±1.4 | 32.5±3.3 | 109.5±6.1 | 4.1±0.3 |
| LT5 | 807±8 | 103.03 | 30.03 |  | 25-35 | 1.1±0.2 | 0.8±0.1 | 3 | 4 | 3 | D | 44.8±2.9 | 4.3±0.6 | 40.1±2.1 | 124.6±3.4 | 4.5±0.1 |
| LT6 | 698±5 | 103.04 | 30.04 |  | 25-35 | 0.8±0.1 | 0.7±0.2 | 4 | 4 | 3 | S | 22.8±3.7 | 3.9±0.9 | 30.8±5.4 | 106.90±1.1 | 5.2±0.2 |
| LT7 | 809±7 | 103.07 | 30.06 |  | 20-30 | 1.1±0.1 | 1.1±0.1 | 3 | 8 | 3 | D | 38.4±5.5 | 4.8±1.7 | 37.3±5.9 | 128.7±1.8 | 4.3±0.2 |

^a^ The data source of tree age is the description of local tea garden owner, not the measured value.

^b^ The number is the number of fertilizer application per year, all fertilizers are compound fertilizers

^c^ The number is the annual frequency of pesticide application.

^d^ The number is annual pruning times.

^e^ S, Single plant planting D, Double plant planting.

^f^ Soil sampling depth is 0-30cm.

**Table S2. The making process of Shihua and Ganlu**

| **The making process of Shihua** | | **The making process of Ganlu** | |
| --- | --- | --- | --- |
| **Process** | **Describe** | **Process** | **Describe** |
| Fixing | Fixing（called shaqingin Chinese）, which step that enzyme inactivation was achieved at high temperatures and short time, using a rotary continuous fixation machine. The fresh leaf variety was "Sichuan middle-leaf tea tree population", and the fresh leaf tenderness was single bud. 6CSQ-60（Changsha Xiangfeng Tea Machinery Manufacturing Co., Ltd） fixing machine was used, and the leaf dosage was 4.0 kg/min. The temperature of the machine was set at 280 ℃ and the leaf temperature was about 80 ± 3 ℃, the roller speed was set at 28 r / min, and the fixing time was 120 ± 5 S. After fixing, the moisture content of the leaf was about 55 ± 5%. | Fixing Ⅰ | The fresh leaf variety was "Sichuan middle-leaf tea tree population", and the fresh leaf tenderness was one bud with one leaf. 6CSQ-60（Changsha Xiangfeng Tea Machinery Manufacturing Co., Ltd） fixing machine was used, and the leaf dosage was 5kg / min. The temperature of the machine was set at 300 ℃ and the leaf temperature was about 80 ℃ ± 5 ℃, the roller speed was set at 35r / min, and the fixing time was 100 ± 5 S。After fixing, the moisture content of the tea was about 50 ± 5%。 |
|  |  | Rolling Ⅰ | Rolling (called rounian in Cinese), a preliminary shaping process of tea leaves. Through the extrusion and pushing by the rolling machine, the tea leaf area was reduced and rolled into strips.6CR-55（Changsha Xiangfeng Tea Machinery Manufacturing Co., Ltd） rolling machine was adopted, the leaf input was 10kg / barrel, and the time was 30 ± 5min。 After rolling, the leaf was tightly rolled into strips, and a small amount of tea juice overflows and sticks to the surface of the attached leaves. At this time, the rate of leaf strips was about 70± 5 %, the rate of cell breakage was about 65 ± 5 %, and the water content of tea leaves was 55 ± 5%。 |
| Shaping | Tea leaves swing rapidly in a certain space through a special shaping machine. This step can make the tea shape straight and even. 6CCB-50 （Changsha Xiangfeng Tea Machinery Manufacturing Co., Ltd）shaping machine was adopted, the temperature was set at 180 ℃ and the leaf temperature was 50 ± 2 ℃, rotating speed 190r / min, leaf volume 2.0kg/pot, shaping time 35 ± 2min。 After shaping, the leaves are flat and straight, the color was green, and the moisture content was about 20 ± 5%. | Fixing Ⅱ | 6CSQ-50 Changsha Xiangfeng Tea Machinery Manufacturing Co., Ltd）fixing machine was used. The temperature was set at 180 ℃ and the leaf temperature was about 55 ± 5 ℃ , the time was 30 ± 5min. After Fixing Ⅱ, the water content of tea leaves was 45 ± 5%. |
|  |  | Rolling Ⅱ | 6CR-55（Changsha Xiangfeng Tea Machinery Manufacturing Co., Ltd） rolling machine was adopted. The amount of leaf was 5kg / barrel and the time was 30 ± 5min. The percentage of sliver formation was about 90%, the percentage of cell breakage was about 85%, and the water content of tea was 45 ± 5%。。 |
|  |  | Fixing Ⅲ | 6CSQ-50（Changsha Xiangfeng Tea Machinery Manufacturing Co., Ltd）fixing machine was used. The temperature was set at 150 ℃ and the leaf temperature was about 50 ± 5 ℃ , the time was100 ± 10S. After Fixing Ⅲ, the water content of tea leaves was 35 ± 5%. |
|  |  | Rolling Ⅲ | Rolling Ⅲ, which step that shape the tea was achieved at low temperatures (40 ℃) and long time(30±5min), using a rotary continuous Shaping machine (6CST-80, Changsha Xiangfeng Tea Machinery Manufacturing Co., Ltd). This was actually a tea shaping process, in order to match with figure 5, we named it Rolling Ⅲ. After drying, the moisture content of tea leaves was 20 ± 1%. |
| Drying | 6CH-16（Changsha Xiangfeng Tea Machinery Manufacturing Co., Ltd） drying machine was used, the temperature was set at 105 ℃ and the time was 20-25min. After drying, the moisture content of tea was 4 ± 1%. | Drying | 6CH-16（Changsha Xiangfeng Tea Machinery Manufacturing Co., Ltd）drying machine was used, the temperature was set at 105 ℃ and the time was 20-25min. After drying, the moisture content of tea was 4 ± 1%. |

**Table S3. The basis for the selection of chemical reference standards in flavor analysis and the interpretation of the flavor vocabulary**

| Type | Flavour | Description^a^ | Reference^b^ |
| --- | --- | --- | --- |
| Aroma | green | Clean and refreshing, aroma similar to grass or fresh leaves | trans-3-hexenol |
|  | tender | The pleasant and delicate aroma of tender raw materials, aroma similar to that of tender corn | nonanal |
|  | chestnut | The aroma is like cooked chestnut | 3-methylbutyraldehyde |
|  | sweet | aroma as heated brown sugar | phenylethanol |
|  | fresh | Fresh and brisk, the fragrance is fresh and pleasant | trans-2-hexenal |
| Taste | sweet | It tastes like sugar and honey | sucrose |
|  | bitter | The root of the tongue after the entrance has been bitter, like bile | quinine |
|  | puckery | Oral cortex or mucous membrane surface contraction, tension or wrinkle, like eating raw persimmon | tannins |
|  | fresh | Fresh and refreshing, like eating beef soup | glutamate |
|  | acid | One of the basic taste sensations, produced by fermentation | citrin |

^a^ Descriptors references national standards: GB / T 14487-2017 tea vascular for sensory evaluation^1^

^b^ Selection of chemical reference standards refers to the FEMA GRAS database（https://www.femaflavour.org）、NIE^2^ and Yau^3^

Reference:

1. General Administration of Quality Supervision, Inspection and Quarantine (AQSIQ) and the Standardization Administration of China (SAC). Tea vascular for sensory evaluation, GB/T 14487-2017. China Standard Publishing House, Beijing (2017).

2. Nie, C. N. et al. Comparison of Different Aroma-Active Compounds of Sichuan Dark Brick Tea (Camellia Sinensis) and Sichuan Fuzhuan Brick Tea Using Gas Chromatography–Mass Spectrometry (GC–MS) and Aroma Descriptive Profile Tests. Eur. Food Res. Technol. 245, (2019).

3. Yau, Njn. & Y. J. Huang. "The effect of membrane-processed water on sensory properties of Oolong tea drinks." Food Quality & Preference. 11,331-339 (2000).

**Table S4. Maximum Residue Limit (MRL), Limit of Detection (LOD) and Limit of Quantitation (LOQ) of 50 pesticide residues(mg/kg)**

| Pesticide | MRL | LOD/LOQ | Pesticide | MRL | LOD | Pesticide | MRL | LOD | Pesticide | MRL | LOD | Pesticide | MRL | LOD |
| --- | --- | --- | --- | --- | --- | --- | --- | --- | --- | --- | --- | --- | --- | --- |
| Paraquat | 0.2 | 0.01/0.03 | Fenpropathrin | 5 | 0.01/0.03 | Omethoate | 0.05 | 0.009/0.025 | Buprofezin | 10 | 0.100/0.250 | Cyfluthrin | 1 | 0.1200/0.4500 |
| Diafenthiuron | 5 | 0.00028/0.0006 | Enazaquin | 15 | 0.005/0.020 | Imidaclothiz | 3 | 0.1/0.3 | Dicofol | 0.2 | 0.016/0.038 | Flucythrinate | 20 | 0.0100/0.0300 |
| Carbofuran | 0.05 | 0.0130/0.041 | Hexachloro-cyclohexane | 0.2 | 0.001/0.003 | Methomyl | 0.2 | 0.01/0.04 | Fenitrothion | 0.5 | 0.02/0.06 | Trichlorfon | 2 | 0.06/0.25 |
| Imidacloprid | 0.5 | 0.0002/0.0006 | Permethrin | 20 | 0.010/0.030 | Methamidophos | 0.5 | 0.005/0.020 | Isocarbophos | 0.05 | 0.0100/0.035 | Pyridaben | 5 | 0.0050/0.0100 |
| pymetrozine | 2 | 0.035/0.140 | Isazofos | 0.01 | 0.010/0.040 | Posfolan-methyl | 0.03 | 0.0005/0.0025 | Terbufos | 0.01 | 0.005/0.018 | Bifenthrin | 5 | 0.0050/0.0200 |
| Acetamiprid | 10 | 0.00036/0.0011 | Ethoprophos | 0.05 | 0.015/0.040 | Indoxacarb | 5 | 0.0075/0.025 | Deltamethrin | 10 | 0.00088/0.00312 | Methamidophos | 0.05 | 0.01/0.03 |
| Thiamethoxam | 10 | 0.0165/0.0330 | Systox | 0.05 | 0.020/0.045 | Phoxim | 0.2 | 0.0207/ | Etoxazole | 15 | 0.0750/0.300 | Phorate | 0.01 | 0.0050/0.0018 |
| Carbendazim | 5 | 0.0005/0.0013 | Fenvalerate | 0.1 | 0.020/0.055 | Endosulfan | 1 | 0.001/0.003 | Acephate | 0.1 | 0.00895/0.02981 | Parathion-methyl | 0.02 | 0.0200/0.0600 |
| Cartap | 20 | 0.52/1.32 | Hexythiazox | 10 | 0.01/0.03 | Cypermethrin | 20 | 0.0150/0.04 | Glyphosate | 1 | 0.0002/0.0006 | Posfolan-methyl | 0.03 | 0.03/0.10 |
| Diflubenzuron | 20 | 0.04/0.13 | Dichloro-diphenyl-trichloroethane | 0.2 | 0.001/0.004 | lamda-cyhalothrin | 15 | 0.005/0.014 | Chlorfenapyr | 20 | 0.0001/0.0004 | Difenoconazole | 10 | 0.0300/0.0850 |
